# Organizational socialization and new employees’ work adaptability in high-tech enterprises: the mediating role of employee proactiveness

**DOI:** 10.3389/fpsyg.2026.1913140

**Published:** 2026-09-14

**Authors:** Shuhua Liu, Chin-Hong Puah

**Affiliations:** 1 Faculty of Economics and Business, Universiti Malaysia Sarawak, Sarawak, Malaysia; 2 Business School, Qilu Institute of Technology, Jinan, Shandong, China

**Keywords:** employee proactiveness, high-tech enterprises, mediating effect, organizational socialization, work adaptability.

## Abstract

Against the backdrop of rapid technological and organizational change in Chinese high-tech enterprises, newcomer adaptation has become increasingly important. This study examines the associations between organizational socialization strategies and new employees’ work adaptability, with particular attention to the mediating role of employee proactiveness. A quantitative cross-sectional design was adopted, and questionnaire data were collected from 419 new employees in Chinese high-tech enterprises. Data were analyzed using SPSS 25.0 and AMOS 26.0, including exploratory factor analysis, confirmatory factor analysis, structural equation modeling, and bootstrapping to examine the mediating role of employee proactiveness. The results showed that scenario, content, and social strategies were positively associated with employee proactiveness. Employee proactiveness was positively associated with performance proficiency, role clarity, and cultural adaptation, but not with interpersonal relationship adaptation. Scenario strategy was positively associated with all four dimensions of work adaptability, whereas content strategy was positively associated with performance proficiency, role clarity, and interpersonal relationship adaptation, but not with cultural adaptation. Social strategy was positively associated only with performance proficiency. Employee proactiveness significantly mediated the associations between organizational socialization strategies and performance proficiency, role clarity, and cultural adaptation, but not interpersonal relationship adaptation. These findings highlight the differential associations between organizational socialization strategies and dimensions of newcomer work adaptability, while underscoring the mediating role of employee proactiveness in several of these associations. By integrating organizational socialization strategies with proactive behavior, the research extends existing organizational socialization literature and provides practical implications for designing differentiated onboarding practices to support newcomer adaptation in high-tech enterprises.

## 1 Introduction

In the twenty-first century, high-tech enterprises have emerged as a critical driving force of global economic development and a strategic pillar enabling countries to overcome the middle-income trap and achieve high-quality sustainable growth (Kou et al., 2023). As major carriers of technological innovation and frontier industrial transformation, high-tech enterprises play an indispensable role in enhancing national innovation capability, facilitating industrial upgrading, and strengthening international competitiveness (Huang et al., 2022; Tu et al., 2023; Zhang et al., 2025). In China, the rapid expansion of high-tech enterprises has generated profound economic and social impacts. By 2024, the number of high-tech enterprises in China had exceeded 500,000, representing an 83% increase compared with 2020 (Qin and Ao, 2025). The industrial clusters and collaborative innovation networks formed by these enterprises have become a key driving force underpinning China’s economic transformation and GDP growth.

High-tech enterprises typically operate in knowledge-intensive and rapidly evolving environments characterized by technological uncertainty, accelerated product iteration, and intensified market competition. Under such conditions, the development of sustainable competitive advantage depends not only on financial investment and technological infrastructure but also on the effective integration and retention of high-quality talent (Wu et al., 2023). Accordingly, talent has become a core strategic resource supporting organizational innovation capability and long-term sustainable development (Liu, 2024). However, in the post-pandemic era, the external environment confronting high-tech enterprises has grown increasingly uncertain, while employees’ expectations regarding career development, organizational support, and work adaptability have continued to rise (Estiana et al., 2025). Against this backdrop, employee turnover has emerged as a major challenge for human resource management and has imposed substantial operational risks and economic costs on high-tech enterprises. Improving newcomers’ work adaptability is therefore critical to mitigating turnover risks, in which organizational socialization plays a vital role.

Existing research has consistently reported a positive association between organizational socialization and newcomer adaptation. Organizational socialization has been associated with employees’ understanding of organizational values, role expectations, and social relationships, as well as with higher organizational commitment and lower turnover intention (Fadahunsi and Olowe, 2025; Bauer et al., 2025). Despite these findings, prior research has primarily examined organizational socialization from a static and outcome-oriented perspective, with limited attention devoted to the theoretical processes through which organizational socialization is associated with employee work adaptability (WA). In particular, the potential mediating role of employee proactiveness has received relatively limited empirical attention, especially in the context of high-tech enterprises (Saks et al., 2011).

Building on Organizational Socialization Theory, Social Cognitive Theory, and the Theory of Work Adjustment, the present study adopts a theory-driven perspective to examine employee work adaptability in organizational settings. Within this perspective, work adaptability is conceptualized as a multidimensional construct reflecting employees’ adjustment to organizational practices, role expectations, interpersonal relationships, and the work environment. Recent research similarly emphasizes that work adaptability can be understood by considering the interrelationships among organizational practices, contextual conditions, and employees’ behavioral engagement (Farías, 2025). Consistent with this perspective, organizational socialization is expected to be positively associated with employees’ acquisition of organizational knowledge, role understanding, and social support, which may, in turn, be associated with higher levels of work adaptability through employee proactiveness.

Accordingly, this study focuses on Chinese high-tech enterprises and proposes a theory-driven conceptual framework linking organizational socialization strategies, employee proactiveness, and work adaptability. Specifically, the study examines the relationships among these constructs, with particular attention to the statistical mediating role of employee proactiveness. By doing so, this study extends the organizational socialization literature by providing empirical evidence regarding the theorized associations among organizational socialization strategies, employee proactiveness, and work adaptability in the context of Chinese high-tech enterprises. Given the cross-sectional design of the study, the proposed framework should be interpreted as a theory-based conceptual model, and the findings reflect theory-driven associations and statistical mediation rather than causal or temporal relationships. Future longitudinal and experimental research is needed to further examine the temporal and causal assumptions underlying the proposed framework.

## 2 Literature review and research hypotheses

### 2.1 Organizational socialization and employee proactiveness

Recent studies increasingly conceptualize employee socialization as an active and reciprocal adjustment process rather than a one-sided organizational intervention (Dimitrova et al., 2023). Social Cognitive Theory (SCT) provides an important theoretical foundation for understanding this process by emphasizing the reciprocal interactions among environmental conditions, individual agency, and behavioral responses (Bandura, 1982, 1986). Existing research further suggests that organizational contextual factors are associated with employees’ psychological resources, motivational states, and behavioral engagement (Wang, 2025).

From a systems perspective, organizational socialization can be understood as an organizational subsystem embedded within broader organizational environments rather than merely a static managerial practice. Within this theoretical perspective, work adaptability is viewed as a process shaped by interactions among organizational structures, interpersonal relationships, individual behaviors, and contextual conditions. During organizational entry, organizational socialization provides employees with informational resources, interpersonal support, and psychological reassurance that may reduce uncertainty and assist their adjustment to new work environments. Structured onboarding practices and clear organizational guidance enable employees to better understand organizational expectations and work requirements, thereby providing conditions that encourage proactive behaviors such as information seeking, feedback inquiry, and work exploration (Ellis et al., 2017). Similarly, supportive interpersonal environments may strengthen employees’ willingness to engage in organizational activities and develop workplace relationships.

The relevance of employee proactiveness is particularly salient in high-tech enterprises characterized by rapid innovation, evolving work demands, and high environmental uncertainty. In such contexts, proactive employees are more likely to seek available resources, explore adaptive approaches, and actively participate in their adjustment process. Nevertheless, limited research has examined how organizational socialization strategies are associated with employee proactiveness within high-tech organizational settings (Mitschelen and Kauffeld, 2025). Therefore, drawing on Organizational Socialization Theory and Social Cognitive Theory, organizational socialization strategies are expected to foster employee proactiveness via distinct organizational pathways (Cable and Parsons, 2001). In this study, organizational socialization strategies are operationalized into three dimensions: scenario strategy (SCS), content strategy (CTS), and social strategy (SS). Accordingly, the following hypotheses are proposed.

H1a: Scenario strategies are positively related to employee proactiveness.

H1b: Content strategies are positively related to employee proactiveness.

H1c: Social strategies are positively related to employee proactiveness.

### 2.2 Employee proactiveness and employee work adaptability

Employee proactiveness (EP) refers to self-initiated, future-oriented behaviors through which individuals actively seek to understand their work environment, acquire relevant information, and improve their ability to accomplish work (Bateman and Crant, 1993). During organizational entry, proactive employees typically engage in behaviors such as information seeking, feedback inquiry, relationship building, and work exploration, which have been widely recognized as important behavioral characteristics of newcomers (Ashforth and Black, 1996). Rather than passively responding to organizational arrangements, proactive employees actively participate in their own adjustment process by identifying available resources and responding to workplace challenges.

The Theory of Work Adjustment (TWA) provides an important theoretical perspective for understanding the relationship between employee proactiveness and work adaptability. According to TWA, work adaptability reflects the correspondence between individual characteristics and work environments and is achieved through continuous interactions between employees and their organizational contexts (Dawis and Lofquist, 1984). Within this perspective, work adaptability is viewed as a multidimensional construct that reflects employees’ adjustment to work, role expectations, interpersonal relationships, and organizational culture rather than as a single organizational outcome.

Previous studies have reported that proactive behaviors are positively associated with newcomer adjustment in organizational settings (Saks et al., 2011). Employees who actively seek information and request feedback are more likely to develop a clearer understanding of organizational expectations and work responsibilities, which may be associated with higher levels of performance proficiency and role clarity (Ellis et al., 2017). Likewise, employees who actively establish workplace relationships may have greater opportunities to obtain informational and social resources that support their interpersonal adjustment. Active exploration of organizational values, norms, and work practices may also contribute to employees’ understanding of organizational culture and facilitate their integration into the organization (Ukawa and Hosoda, 2023).

These relationships are particularly relevant in high-tech enterprises, where employees are frequently required to respond to rapidly changing technologies, knowledge-intensive work, and uncertain work environments (Akkermans et al., 2024). Under such conditions, employee proactiveness represents an important individual behavioral resource that may be associated with different dimensions of work adaptability. Nevertheless, empirical evidence regarding these relationships among newcomers in Chinese high-tech enterprises remains relatively limited. Examining these relationships may therefore contribute to a more comprehensive understanding of newcomer adjustment within knowledge-intensive organizational contexts.

Following Chan and Schmitt (2000), this study conceptualizes work adaptability as comprising four dimensions: performance proficiency (PP), role clarity (RC), interpersonal relationships (IR), and cultural adaptation (CA). Based on the Theory of Work Adjustment and previous research on newcomer proactivity, employee proactiveness is expected to be positively related to each dimension of work adaptability. Accordingly, the following hypotheses are proposed:

H2a: Employee proactiveness is positively related to performance proficiency.

H2b: Employee proactiveness is positively related to role clarity.

H2c: Employee proactiveness is positively related to interpersonal relationships.

H2d: Employee proactiveness is positively related to cultural adaptation.

### 2.3 Organizational socialization and employee work adaptability

Organizational socialization is widely recognized as an important organizational process through which newcomers acquire the knowledge, role expectations, and social competencies necessary to function effectively within an organization (Chao et al., 1994; Schein, 1968). According to Organizational Socialization Theory (OST), structured socialization practices provide informational, relational, and organizational resources that support newcomers’ understanding of work requirements and organizational norms during organizational entry (Jones, 1986). Rather than representing a one-time onboarding activity, organizational socialization reflects a continuous process through which newcomers become familiar with organizational expectations and workplace practices.

Drawing on the Theory of Work Adjustment (TWA), work adaptability reflects the correspondence between employees and their work environments across multiple dimensions, including performance proficiency, role clarity, interpersonal relationships, and cultural adaptation (Chan and Schmitt, 2000; Dawis and Lofquist, 1984). Organizational socialization strategies may therefore provide organizational resources that are theoretically associated with these dimensions of work adaptability.

In this study, organizational socialization is operationalized through scenario strategies, content strategies, and social strategies. Scenario strategies emphasize structured work arrangements and role clarification, which may be associated with employees’ understanding of job responsibilities and work expectations. Content strategies focus on providing organizational knowledge, work procedures, and work-related information that may support employees’ acquisition of role-related knowledge and organizational understanding. Social strategies emphasize interpersonal interaction, communication, and organizational support, providing newcomers with opportunities to establish workplace relationships and become familiar with organizational culture (Gardner et al., 2022).

Previous studies have reported positive associations between organizational socialization and various employee outcomes, including organizational commitment, job satisfaction, work performance, and newcomer adjustment (Akkermans et al., 2024; Gardner et al., 2022). However, empirical evidence examining how different organizational socialization strategies are associated with multiple dimensions of work adaptability remains relatively limited, particularly in Chinese high-tech enterprises. Addressing this gap may contribute to a more comprehensive understanding of newcomer adaptation within knowledge-intensive organizational settings.

Based on Organizational Socialization Theory and the Theory of Work Adjustment, this study proposes that each dimension of organizational socialization strategies is positively related to each dimension of work adaptability. Accordingly, the following hypotheses are proposed:

H3a: Scenario strategies are positively related to performance proficiency.

H3b: Scenario strategies are positively related to role clarity.

H3c: Scenario strategies are positively related to interpersonal relationships.

H3d: Scenario strategies are positively related to cultural adaptation.

H3e: Content strategies are positively related to performance proficiency.

H3f: Content strategies are positively related to role clarity.

H3g: Content strategies are positively related to interpersonal relationships.

H3h: Content strategies are positively related to cultural adaptation.

H3i: Social strategies are positively related to performance proficiency.

H3j: Social strategies are positively related to role clarity.

H3k: Social strategies are positively related to interpersonal relationships.

H3l: Social strategies are positively related to cultural adaptation.

### 2.4 The mediating role of employee proactiveness

Although previous studies have separately examined organizational socialization, employee proactiveness, and work adaptability, relatively limited research has integrated these constructs within a single theoretical framework, particularly in the context of high-tech enterprises (Mumpuni and Handayani, 2024). Drawing on Social Cognitive Theory (SCT), organizational environments are associated with employees’ behavioral responses through reciprocal interactions among environmental conditions, individual cognition, and behavioral engagement (Bandura, 1986). Within this perspective, organizational socialization strategies may provide informational, relational, and organizational resources that support newcomers’ proactive engagement during organizational entry.

Employee proactiveness reflects self-initiated behaviors through which newcomers actively seek information, request feedback, establish workplace relationships, and acquire organizational knowledge (Ashforth and Black, 1996; Saks et al., 2011). These behaviors are theoretically associated with employees’ understanding of work requirements, role expectations, interpersonal interactions, and organizational culture, which together represent important dimensions of work adaptability.

In high-tech enterprises, where employees frequently encounter rapidly changing technologies, complex work, and uncertain organizational environments, proactive behavioral engagement may represent an important pathway through which organizational socialization strategies are associated with newcomer work adaptability. Nevertheless, empirical evidence regarding this mediating relationship remains limited, particularly in Chinese high-tech enterprises.

Based on Organizational Socialization Theory, Social Cognitive Theory, and the Theory of Work Adjustment, this study proposes that employee proactiveness statistically mediates the relationships between organizational socialization strategies and work adaptability. Accordingly, the following hypotheses are proposed:

H4a: Employee proactiveness mediates the relationship between scenario strategy and performance proficiency

H4b: Employee proactiveness mediates the relationship between scenario strategy and role clarity

H4c: Employee proactiveness mediates the relationship between scenario strategy and interpersonal relationships

H4d: Employee proactiveness mediates the relationship between scenario strategy and cultural adaptation

H4e: Employee proactiveness mediates the relationship between content strategy and performance proficiency

H4f: Employee proactiveness mediates the relationship between content strategy and role clarity

H4g: Employee proactiveness mediates the relationship between content strategy and interpersonal relationships

H4h: Employee proactiveness mediates the relationship between content strategy and cultural adaptation

H4i: Employee proactiveness mediates the relationship between social strategy and performance proficiency

H4j: Employee proactiveness mediates the relationship between social strategy and role clarity

H4k: Employee proactiveness mediates the relationship between social strategy and interpersonal relationships

H4l: Employee proactiveness mediates the relationship between social strategy and cultural adaptation

### 2.5 Research framework

This study integrates Organizational Socialization Theory, Social Cognitive Theory, and the Theory of Work Adjustment into a theory-informed conceptual framework to examine how organizational contextual factors and employee proactive behaviors are associated with work adaptability outcomes. Based on the preceding theoretical discussions, this study proposes a conceptual model linking organizational socialization strategies, employee proactiveness, and work adaptability.

Specifically, organizational socialization strategies are expected to be directly associated with work adaptability and indirectly associated with work adaptability through employee proactiveness. The proposed framework represents a process-oriented conceptual model that illustrates the theoretically proposed relationships among organizational practices, employee behavioral responses, and adaptation outcomes within high-tech organizational contexts (Figure 1).

**FIGURE 1:**
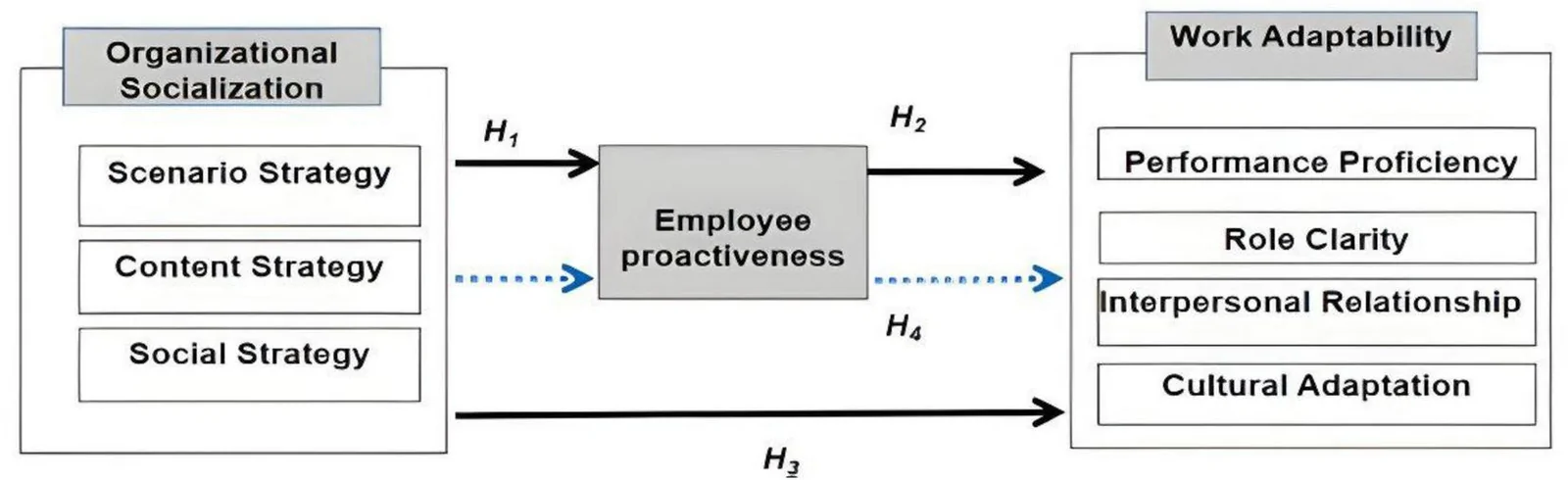
Research framework. Solid lines represent hypothesized direct relationships; dashed lines represent the indirect relationship through employee proactiveness. H1: Organizational socialization strategies (scenario, content, and social strategies) are positively related to employee proactiveness; H2: Employee proactiveness is positively related to new employees’ work adaptability (including performance proficiency, role clarity, interpersonal relationships, and cultural adaptation); H3: Organizational socialization strategies are positively related to new employees’ work adaptability; H4: Employee proactiveness mediates the relationship between organizational socialization strategies and new employees’ work adaptability.

## 3 Materials and methods

### 3.1 Research design

This study employed a quantitative cross-sectional design to examine the relationships among organizational socialization, employee proactiveness, and work adaptability. Data were collected using a structured self-administered questionnaire and analyzed with SPSS 25.0 and AMOS 26.0. Descriptive statistics, confirmatory factor analysis (CFA), structural equation modeling (SEM), and bootstrap mediation analysis were conducted.

Organizational socialization (scenario strategy, content strategy, and social strategy) was specified as the independent variable, employee proactiveness as the mediating variable, and work adaptability (performance proficiency, role clarity, interpersonal relationships, and cultural adaptation) as the dependent variable. Demographic variables, including age and organizational tenure, were collected and incorporated as control variables in the structural model to account for their potential influence on employee work adaptability. Other demographic characteristics were collected and reported solely for sample description.

### 3.2 Research participants

Jinan is one of China’s major high-tech industrial hubs, hosting numerous nationally recognized high-tech enterprises across software, electronic information, and intelligent manufacturing sectors. The target population consisted of employees from high-tech enterprises in Jinan, China. The participating enterprises were selected based on their official recognition as high-tech enterprises under China’s High-Tech Enterprise Recognition and Management framework, which emphasizes technological innovation capability, research and development activities, and knowledge-intensive business operations. Data were collected between June and August 2024 from three high-tech enterprises operating in the software, electronic information, and intelligent manufacturing sectors.

The selected enterprises represent different ownership structures, organizational sizes, and high-tech industry categories, providing a suitable context for examining newcomer work adaptability. Questionnaires were distributed online through internal company platforms, and participation was anonymous, voluntary, and confidential. A total of 450 questionnaires were distributed. After applying the screening criteria, 31 invalid questionnaires were excluded from the 450 collected responses. Specifically, 8 responses were removed because respondents did not meet the eligibility criteria, 4 contained substantial missing data ( > 10% unanswered items), 9 were completed within less than 15 min, 3 showed inconsistent responses to screening questions, 5 exhibited repetitive or straight-line response patterns, and 2 were identified as duplicate submissions through the survey platform. Therefore, 419 valid questionnaires were retained for subsequent analysis. The final sample size was considered adequate for structural equation modeling analysis (Bonds-Raacke, 2019; Wolf et al., 2013).

Given that organizational socialization is a continuous adjustment process rather than a short-term event limited to the initial entry period, this study adopted a broader definition of newcomers. Specifically, employees with organizational tenure of 3 years or less were included as newcomers (He et al., 2020). This operational criterion is appropriate for identifying employees who are still likely to experience organizational socialization and adjustment processes, particularly in high-tech enterprises characterized by continuous learning and technological change (Van Maanen and Schein, 1979; Morrison, 1993; Bauer et al., 2007; Saks and Gruman, 2018).

### 3.3 Research measures

All variables were measured using a 5-point Likert scale (1 = strongly disagree, 5 = strongly agree), with higher scores representing higher levels of the construct. All scales were adapted from well-established international instruments and appropriately revised to fit the Chinese organizational context.

Organizational socialization was measured based on Jones (1986) organizational socialization tactics framework. The original six tactics were grouped into three dimensions: scenario strategy (collective and formal tactics), content strategy (sequential and fixed tactics), and social strategy (serial and investiture tactics), representing contextual, informational, and relational mechanisms of newcomer socialization. Employee proactiveness was measured using Frese et al.’s (1997) proactive behavior scale, consisting of seven items assessing self-initiated problem-solving, opportunity identification, and work improvement behaviors. Work adaptability was measured drawing on Morrison’s (1993) foundational work on newcomer adjustment and relevant scholarship on organizational entry adaptation (Chan and Schmitt, 2000). The construct covers four dimensions: performance proficiency, role clarity, interpersonal relationships, and cultural adaptation. Performance proficiency and role clarity were adapted from Morrison (1993) and Bauer et al. (2007). Interpersonal relationships originate from studies of interpersonal integration (Chao et al., 1994; Morrison, 1993), while cultural adaptation draws on research regarding organizational culture assimilation (Chao et al., 1994).

To ensure measurement equivalence, the questionnaire items were translated into Chinese and back-translated into English by bilingual researchers. The adapted items were further reviewed by experts in organizational behavior and human resource management and refined based on a pilot test involving 45 new employees from three high-tech enterprises. Reliability and construct validity were assessed through Cronbach’s alpha, composite reliability (CR), average variance extracted (AVE), exploratory factor analysis (EFA), and confirmatory factor analysis (CFA).

## 4 Data analysis and results

### 4.1 Data screening and preliminary analysis

Statistical analyses were conducted using SPSS 25.0 and AMOS 26.0. SPSS 25.0 was used for data screening and preliminary analyses, including the examination of missing values and outliers, descriptive statistics, and Pearson correlation analyses. The final sample consisted of 419 valid responses. The demographic characteristics of the respondents, including gender, age, educational attainment, number of previous employers, and organizational tenure, are presented in Table 1.

**TABLE 1:** Participant demographics.

Characteristics	Choice	Frequency	Percent
Gender	Male	285	68
	Female	134	32
Age	Less than 25 years	36	8.6
	26–30	175	41.7
	31–35	170	40.6
	36–40	30	7.2
	More than 40 years	8	1.9
Education	Below undergraduate level	41	9.8
	Undergraduate degree (or equivalent)	274	65.4
	Postgraduate level or above (or equivalent)	104	24.8
Marital	Married	185	44.2
	Unmarried	203	48.4
	Divorced	29	6.9
	Widowed	2	0.5
Number of previous employers	0	7	1.7
	1	184	43.9
	2–3	221	52.7
	4 or more	7	1.7
	Less than 3 months	24	5.7
Tenure	3–6 months	87	20.8
	7–12 months	136	32.5
	13–24 months	88	21
	25–36 months	84	20

Descriptive statistics and Pearson correlation analyses were conducted using SPSS 25.0, and the results are presented in Table 2. The mean scores of the study constructs ranged from 4.176 to 4.298, with standard deviations ranging from 0.515 to 0.620. Pearson correlation analyses indicated that all study constructs were positively and significantly correlated (*p* < 0.001), with correlation coefficients ranging from 0.388 to 0.665. These findings provide an overview of the distributions and bivariate associations among the study constructs prior to the subsequent measurement and structural model analyses.

**TABLE 2:** Descriptive statistics and correlation matrix of study variables.

Variable	M	SD	SCS	CTS	SS	EP	PP	RC	IR	CA
SCS	4.298	0.563	1	1	1	1	1	1	1	1
CTS	4.279	0.556	0.628***							
SS	4.284	0.555	0.629***	0.665***						
EP	4.247	0.540	0.613***	0.607***	0.628***					
PP	4.285	0.515	0.621***	0.622***	0.628***	0.640***				
RC	4.198	0.555	0.492***	0.502***	0.483***	0.506***	0.582***			
IR	4.196	0.620	0.433***	0.447***	0.396***	0.397***	0.475***	0.466***		
CA	4.176	0.583	0.546***	0.517***	0.516***	0.549***	0.554***	0.491***	0.388***	

SCS, Scenario Strategy; CTS, Content Strategy; SS, Social Strategy; EP, Employee Proactiveness; PP, Performance Proficiency; RC, Role Clarity; IR, Interpersonal relationships; CA, Cultural Adaptation. *p < 0.05, **p < 0.01, ***p < 0.001.

Subsequently, confirmatory factor analysis (CFA) was performed in AMOS to assess the measurement model and examine convergent and discriminant validity. Discriminant validity was evaluated using the Fornell–Larcker criterion, whereby the square root of the average variance extracted (AVE) for each construct exceeded its correlations with other constructs. Based on the validated measurement model, structural equation modeling (SEM) was conducted to test the hypothesized relationships among variables. In addition, the Bootstrap method with 5,000 resamples was employed to examine the significance of indirect effects and verify the robustness of the mediation results. Given that this study aimed to test theoretically established relationships among latent constructs and assess the overall model fit, covariance-based SEM (CB-SEM) using AMOS 26.0 was selected as the appropriate analytical approach (Byrne, 2016; Hair et al., 2019).

### 4.2 Reliability and validity analysis

#### 4.2.1 Reliability Analysis

Table 3 presents the results of the reliability analysis. The Cronbach’s α coefficients for all variables in this study fall within the acceptable range, indicating that the scales possess satisfactory internal consistency reliability.

**TABLE 3:** Reliability analysis.

Variables	Number of items	Cronbach’s α
SCS	4	0.906
CTS	4	0.893
SS	4	0.885
EP	7	0.943
PP	4	0.857
RC	4	0.892
IR	3	0.904
CA	4	0.811

#### 4.2.2 Common method bias test

To evaluate common method bias (CMB), the Common Latent Factor (CLF) approach was adopted following Podsakoff et al. (2003), Williams et al. (2010), and Fuller et al. (2016). A common latent factor was incorporated into the baseline CFA model while the original measurement structure was preserved, with the variance of this latent factor freely estimated. Standardized item loadings were compared between the baseline model and the CLF-augmented model, and absolute loading differences were calculated. As shown in Table 4, except for χ^2^/df, all differences were below 0.10, suggesting no substantial changes in the measurement relationships.

**TABLE 4:** Fit indices of the baseline CFA and common-latent-factor-augmented CFA models.

Model	χ ^2^/df	RMSEA	CFI	TLI	IFI	PGFI	PNFI	PCFI
Original CFA model	2.666	0.063	0.927	0.918	0.928	0.703	0.791	0.825
CFA model with common latent factor	2.409	0.058	0.939	0.931	0.936	0.709	0.799	0.833

To assess common method bias (CMB), Harman’s single-factor test was conducted using an unrotated principal component analysis. The first factor accounted for 46.148% of the total variance, below the commonly used 50% threshold (Podsakoff et al., 2003). In addition, a Common Latent Factor (CLF) was incorporated into the baseline CFA model while retaining the original theoretical measurement structure, with all 34 measurement items loading simultaneously on their respective theoretical constructs and the global CLF. The CLF was specified as uncorrelated with the theoretical constructs, and its variance was freely estimated. Standardized loadings from the baseline and CLF models were compared, with all absolute differences below 0.20, indicating no substantial changes in the measurement loadings. Overall, the results did not indicate that a single method factor dominated the variance of the variables (Podsakoff et al., 2012).

#### 4.2.3 Multicollinearity assessment

Prior to conducting structural equation modeling (SEM), multicollinearity diagnostics were performed to examine whether excessive correlations existed among predictor variables. This assessment was particularly important because the three dimensions of organizational socialization tactics (SCS, CTS, and SS) represent conceptually related constructs and may demonstrate moderate correlations. Multicollinearity was assessed using tolerance values and variance inflation factors (VIF), with tolerance values above 0.20 and VIF values below 5.0 considered acceptable thresholds. As shown in Table 5, all tolerance values ranged from 0.481 to 0.930, while all VIF values ranged from 1.075 to 2.079, indicating that multicollinearity was not a concern among the predictor variables.

**TABLE 5:** Multicollinearity assessment.

Variable	Tolerance	VIF
CTS	0.481	2.078
SS	0.481	2.079
SCS	0.522	1.917
Age dummy 1 ( ≤ 30 years)	0.885	1.13
Age dummy 2 ( ≥ 36 years)	0.872	1.147
Tenure dummy 1 (13–24 months)	0.93	1.075
Tenure dummy 2 (25–36 months)	0.897	1.114

For age, respondents aged 31–35 years served as the reference group. Age Dummy 1 represented respondents aged 30 years or below, whereas Age Dummy 2 represented respondents aged 36 years or above. For organizational tenure, respondents with a tenure of 12 months or less served as the reference group. Tenure Dummy 1 represented respondents with 13–24 months of organizational tenure, whereas Tenure Dummy 2 represented respondents with 25–36 months of organizational tenure.

#### 4.2.4 Confirmatory factor analysis

Confirmatory factor analysis (CFA) was conducted to assess the reliability and validity of the measurement model. Following previous studies (Fornell and Larcker, 1981; Henseler et al., 2015; Chong et al., 2014), AMOS 26.0 was employed for model evaluation. As shown in Table 6, the measurement model demonstrated an acceptable fit (χ^2^/df = 2.666, RMSEA = 0.063, GFI = 0.839, AGFI = 0.808, IFI = 0.928, TLI = 0.918, and CFI = 0.927). Although GFI and AGFI were below the conventional threshold of 0.90, model fit should be evaluated using multiple indices rather than a single criterion (Hu and Bentler, 1999). Given the satisfactory values of χ/^2^df, RMSEA, IFI, TLI, and CFI, the measurement model was considered acceptable for subsequent structural analysis. Additionally, the eight-factor model showed substantially better fit than the single-factor model (χ^2^/df = 9.200, RMSEA = 0.140, CFI = 0.622), supporting the discriminant validity of the constructs.

**TABLE 6:** Confirmatory factor analysis results.

Model name	χ ^2^/df	RMSEA	CFI	TLI	IFI	PGFI	PNFI	PCFI	RMR
Single-factor model	9.200	0.140	0.622	0.597	0.623	0.479	0.559	0.584	0.036
Three-factor model	6.753	0.117	0.736	0.718	0.737	0.559	0.658	0.668	0.030
Six-factor model	4.067	0.086	0.862	0.849	0.863	0.649	0.756	0.790	0.025
Seven-factor model A	3.559	0.078	0.887	0.874	0.887	0.660	0.776	0.800	0.022
Seven-factor model B	3.261	0.074	0.900	0.889	0.900	0.679	0.778	0.812	0.022
Seven-factor model C	3.513	0.078	0.889	0.877	0.889	0.661	0.768	0.802	0.022
Eight-factor model	2.666	0.063	0.927	0.918	0.928	0.703	0.791	0.825	0.021

Alternative models were specified by combining theoretically related dimensions to evaluate discriminant validity. The single-factor model assumes that all observed indicators load onto one latent construct. The three-factor model includes OSS, EP and WA. The six-factor model consists of OSS, EP, PP, RC, IR, and CA. The seven-factor models are specified as follows: Model A combines SCS and CTS; Model B combines SCS and SS; Model C combines CTS and SS, while retaining EP, PP, RC, IR, and CA as distinct factors. The eight-factor baseline model includes SCS, CTS, SS, EP, PP, RC, IR, and CA as separate latent constructs.

#### 4.2.5 Convergent validity

Convergent validity and internal consistency reliability were examined using factor loadings (FL), composite reliability (CR), and average variance extracted (AVE). The results showed that all factor loadings ranged from 0.753 to 0.961, exceeding the recommended threshold of 0.70. In addition, the CR values ranged from 0.859 to 0.946, while the AVE values ranged from 0.604 to 0.773, both surpassing the recommended criteria of 0.70 and 0.50, respectively. These findings (see Table 7) indicate satisfactory internal consistency reliability and convergent validity for all constructs, supporting the adequacy of the measurement model for subsequent analyses.

**TABLE 7:** Standardized factor loadings, CR, and AVE.

Construct	Item	Standardized factor Loadings	CR	AVE
SCS	SCS1	0.762	0.910	0.717
	SCS2	0.846		
	SCS3	0.905		
	SCS4	0.867		
CTS	CTS1	0.775	0.895	0.682
	CTS2	0.809		
	CTS3	0.885		
	CTS4	0.831		
SS	SS1	0.804	0.890	0.669
	SS2	0.850		
	SS3	0.847		
	SS4	0.769		
EP	EP1	0.835	0.946	0.713
	EP2	0.853		
	EP3	0.874		
	EP4	0.845		
	EP5	0.857		
	EP6	0.852		
	EP7	0.791		
PP	PP1	0.760	0.859	0.604
	PP2	0.755		
	PP3	0.786		
	PP4	0.806		
RC	RC1	0.799	0.896	0.684
	RC2	0.870		
	RC3	0.880		
	RC4	0.753		
IR	IR1	0.839	0.911	0.773
	IR2	0.961		
	IR3	0.832		
CA	CA1	0.800	0.885	0.659
	CA2	0.828		
	CA3	0.848		
	CA4	0.769		

#### 4.2.6 Discriminant validity

Table 8 reports the results of discriminant validity based on the Fornell–Larcker criterion. The square root of the average variance extracted (AVE) of each construct is larger than its correlation coefficients with other constructs, indicating satisfactory discriminant validity.

**TABLE 8:** Discriminant validity (Fornell–Larcker Criterion).

Variable	SCS	CTS	SS	EP	PP	RC	IR	CA
SCS	0.847	0.826	0.818	0.844	0.777	0.827	0.879	0.812
CTS	0.678							
SS	0.684	0.733						
EP	0.638	0.649	0.674					
PP	0.687	0.700	0.713	0.699				
RC	0.513	0.526	0.506	0.523	0.637			
IR	0.432	0.461	0.404	0.412	0.518	0.478		
CA	0.590	0.573	0.557	0.582	0.625	0.520	0.397	

### 4.3 Structural model assessment

In this study, a structural equation model (SEM) was employed to verify the proposed variable relationships. Exogenous variables were included in the analysis to examine endogenous variables using AMOS 26.0, and to determine whether linear relationships existed between independent and dependent variables. As shown in Table 9, the structural fitness of the research model showed an acceptable level of model fit (RMSEA = 0.051 < 0.080, CFI = 0.941 > 0.90, Chisq/df = 2.086 < 3) (Byrne, 2016).

**TABLE 9:** Model fitting results.

Indicators to be fitted	Critical value	Recommended criterion	Result
Chi-square		1310.305	
Degrees of freedom (df)		628	
χ^2^/df	< 3	2.086	Y
RMSEA	< 0.10	0.051	Y
GFI	> 0.8	0.857	Y
AGFI	> 0.8	0.831	Y
IFI	> 0.9	0.941	Y
TLI	> 0.9	0.934	Y
CFI	> 0.9	0.941	Y

### 4.4 Mediation analysis

This study adopted the bootstrapping method with 5,000 resamples to examine the mediating effect of employee proactiveness between organizational socialization strategies and employee work adaptability. The empirical results reveal that employee proactiveness exerts significant mediating effects in most research paths. The mediation analysis results are presented in Table 10. Employee proactiveness partially mediated the associations between scenario strategy and performance proficiency, role clarity, and cultural adaptation, but showed no significant mediation effect for interpersonal relationships. For content strategy, employee proactiveness partially mediated its associations with performance proficiency and role clarity, fully mediated its association with cultural adaptation, and showed no significant mediation effect for interpersonal relationships. In terms of social strategy, employee proactiveness partially mediates its correlation with performance proficiency, and fully mediates its relationships with role clarity and cultural adaptation. Collectively, none of the indirect effects on interpersonal relationship adaptation reached statistical significance at the 0.05 level. These findings indicate that individual employee proactiveness effectively facilitates employees’ oriented and cognitive work adaptation, while having no prominent effect on interpersonal relationships. This finding suggests that interpersonal relationships may involve additional contextual factors beyond individual proactive behaviors, such as interpersonal interactions and team climate.

**TABLE 10:** Mediating effect test.

Path	Total effect (B.)	95%		*P*	Direct effect (B.)	95%		*P*	Indirect effect (B.)	95%		*P*	Mediation type
		LB	UP			LB	UP			LB	UP		
SCS → EP → PP	0.285	0.137	0.429	0.001	0.219	0.074	0.361	0.004	0.066	0.029	0.125	0.001	Partial Mediation
SCS → EP → RC	0.26	0.073	0.463	0.008	0.207	0.029	0.397	0.025	0.053	0.014	0.116	0.005	Partial mediation
SCS → EP → IR	0.251	0.066	0.458	0.011	0.206	0.013	0.414	0.039	0.045	−0.001	0.120	0.054	No mediation
SCS → EP → CA	0.367	0.187	0.546	0.001	0.294	0.119	0.478	0.002	0.073	0.033	0.139	0.001	Partial mediation
CTS → EP → PP	0.275	0.127	0.432	0.001	0.216	0.062	0.373	0.007	0.059	0.020	0.118	0.002	Partial mediation
CTS → EP → RC	0.276	0.059	0.498	0.010	0.229	0.008	0.456	0.043	0.047	0.012	0.114	0.005	Partial mediation
CTS → EP → IR	0.342	0.139	0.562	0.001	0.302	0.089	0.529	0.006	0.04	−0.001	0.110	0.060	No mediation
CTS → EP → CA	0.266	0.062	0.470	0.015	0.201	−0.008	0.409	0.060	0.065	0.021	0.137	0.002	Full mediation
SS → EP → PP	0.279	0.125	0.431	0.001	0.206	0.05	0.362	0.009	0.072	0.033	0.137	0.001	Partial mediation
SS → EP → RC	0.159	−0.033	0.372	0.105	0.101	−0.106	0.320	0.334	0.058	0.017	0.130	0.004	Full mediation
SS → EP → IR	0.078	−0.132	0.299	0.456	0.028	−0.194	0.265	0.769	0.049	−0.002	0.126	0.064	No mediation
SS → EP → CA	0.181	−0.009	0.392	0.062	0.101	−0.094	0.304	0.273	0.08	0.033	0.162	0.001	Full mediation

Est, Estimate; LB, Lower Bound of 95% confidence interval; UP, Upper Bound of 95% confidence interval. Partial mediation indicates that both direct and indirect effects were significant. Full mediation indicates that the indirect effect was statistically significant, whereas the direct effect was not statistically significant after the mediator was included in the model. No mediation indicates that the indirect effect was not significant based on the bootstrapping results.

Overall, the findings suggest that organizational socialization strategies are associated with employees’ work adaptability indirectly through employee proactiveness. However, the proposed mediating mechanism appears to be more evident for work-related and cognitive adaptation outcomes than for interpersonal relationship adaptation, which may involve additional relational and contextual factors.

### 4.5 Hypothesis testing results

This study adopted the bootstrapping method to estimate standard errors and *p*-values and examine the significance of structural path coefficients. Age and tenure were included as control variables in the structural model to account for their potential effects on work adaptability. The results of hypothesis testing are summarized in Table 11.

**TABLE 11:** Results of hypothesis testing and control variables.

Hypothesis	Path	B	S.E.	C.R.	ββ	Wald *P*	95% CI (BC)		Decision
							Lower	Upper	
Direct effects (H1–H3)									
H1a	SCS → EP	0.293	0.070	4.162	0.259	<0.001	0.131	0.448	Supported
H1b	CTS → EP	0.263	0.075	3.491	0.241	<0.001	0.100	0.423	Supported
H1c	SS → EP	0.324	0.074	4.345	0.308	<0.001	0.184	0.497	Supported
H2a	EP → PP	0.223	0.050	4.426	0.258	<0.001	0.112	0.338	Supported
H2b	EP → RC	0.180	0.066	2.742	0.188	0.006	0.047	0.333	Supported
H2c	EP → IR	0.152	0.077	1.988	0.139	0.047	−0.025	0.328	Rejected
H2d	EP → CA	0.248	0.066	3.735	0.244	<0.001	0.113	0.390	Supported
H3a	SCS → PP	0.219	0.061	3.601	0.224	<0.001	0.074	0.361	Supported
H3b	SCS → RC	0.207	0.080	2.582	0.191	0.010	0.029	0.397	Supported
H3c	SCS → IR	0.206	0.094	2.192	0.166	0.028	0.013	0.414	Supported
H3d	SCS → CA	0.294	0.081	3.625	0.255	<0.001	0.119	0.478	Supported
H3e	CTS → PP	0.216	0.065	3.309	0.229	<0.001	0.062	0.373	Supported
H3f	CTS → RC	0.229	0.086	2.646	0.219	0.008	0.008	0.456	Supported
H3g	CTS → IR	0.302	0.101	2.991	0.253	0.003	0.089	0.529	Supported
H3h	CTS → CA	0.201	0.086	2.334	0.182	0.020	−0.008	0.409	Rejected
H3i	SS → PP	0.206	0.066	3.151	0.227	0.002	0.050	0.362	Supported
H3j	SS → RC	0.101	0.085	1.180	0.100	0.238	−0.106	0.320	Rejected
H3k	SS → IR	0.028	0.100	0.285	0.025	0.775	−0.194	0.265	Rejected
H3l	SS → CA	0.101	0.085	1.181	0.095	0.238	−0.094	0.304	Rejected
Indirect effects (H4)									
H4a	SCS → EP → PP	0.066	0.024	–	–	–	0.029	0.125	Supported
H4b	SCS → EP → RC	0.053	0.026	–	–	–	0.014	0.116	Supported
H4c	SCS → EP → IR	0.045	0.030	–	–	–	−0.001	0.120	Rejected
H4d	SCS → EP → CA	0.073	0.026	–	–	–	0.033	0.139	Supported
H4e	CTS → EP → PP	0.059	0.024	–	–	–	0.020	0.118	Supported
H4f	CTS → EP → RC	0.047	0.025	–	–	–	0.012	0.114	Supported
H4g	CTS → EP → IR	0.040	0.028	–	–	–	−0.001	0.110	Rejected
H4h	CTS → EP → CA	0.065	0.029	–	–	–	0.021	0.137	Supported
H4i	SS → EP → PP	0.072	0.025	–	–	–	0.033	0.137	Supported
H4j	SS → EP → RC	0.058	0.028	–	–	–	0.017	0.130	Supported
H4k	SS → EP → IR	0.049	0.032	–	–	–	−0.002	0.126	Rejected
H4l	SS → EP → CA	0.080	0.031	–	–	–	0.033	0.162	Supported
Control variables									
–	AGE1 → PP	−0.035	0.033	−1.071	−0.04	0.284	−0.101	0.029	Not significant
–	AGE2 → PP	0.020	0.057	0.354	0.013	0.723	−0.093	0.136	Not significant
–	TEN1 → PP	−0.035	0.039	−0.891	−0.033	0.373	−0.113	0.042	Not significant
–	TEN2 → PP	0.001	0.041	0.035	0.001	0.972	−0.084	0.087	Not significant
–	AGE1 → RC	−0.009	0.043	−0.214	−0.01	0.83	−0.094	0.08	Not significant
–	AGE2 → RC	0.039	0.076	0.513	0.023	0.608	−0.104	0.193	Not significant
–	TEN1 → RC	0.009	0.052	0.172	0.008	0.864	−0.095	0.110	Not significant
–	TEN2 → RC	0.010	0.054	0.190	0.009	0.850	−0.099	0.114	Not significant
–	AGE1 → IR	−0.051	0.051	−1.010	−0.047	0.313	−0.158	0.048	Not significant
–	AGE2 → IR	0.065	0.089	0.734	0.034	0.463	−0.109	0.222	Not significant
–	TEN1 → IR	−0.099	0.061	−1.634	0.074	0.102	−0.223	0.017	Not significant
–	TEN2 → IR	−0.147	0.064	−2.313	−0.107	0.021	−0.274	−0.023	Significant
–	AGE1 → CA	0.010	0.044	0.221	0.009	0.825	−0.083	0.093	Not significant
–	AGE2 → CA	0.088	0.076	1.153	0.050	0.249	−0.064	0.231	Not significant
–	TEN1 → CA	0.020	0.052	0.379	0.016	0.705	−0.078	0.117	Not significant
–	TEN2 → CA	−0.111	0.054	−2.047	−0.088	0.041	−0.23	0.009	Not significant

B, unstandardized coefficient; S.E, standard error; C.R, critical ratio; β, standardized coefficient; CI, confidence interval; BC, bias-corrected. Hypotheses were evaluated based on the 95% bias-corrected bootstrap confidence intervals. Age and tenure were included as control variables and were not associated with specific hypotheses. “–” indicates that the statistic was not applicable or not reported.

The results showed that all three dimensions of organizational socialization strategies were significantly associated with employee proactiveness, supporting H1a–H1c. Employee proactiveness showed positive links to performance proficiency, role clarity, and cultural adaptation, whereas its effect on interpersonal relationship adaptation was not supported. Regarding the direct effects of organizational socialization strategies on work adaptability, most hypothesized relationships were supported. However, social strategy did not significantly predict role clarity, interpersonal relationship adaptation, or cultural adaptation, and content strategy did not significantly predict cultural adaptation.

The mediation analysis indicated that employee proactiveness significantly mediated most relationships between organizational socialization strategies and work adaptability dimensions. Specifically, the indirect effects were significant for performance proficiency, role clarity, and cultural adaptation, whereas none of the indirect effects on interpersonal relationship adaptation reached statistical significance. These findings suggest that employee proactiveness serves as an important mechanism linking organizational socialization strategies with work-related and cognitive adaptation outcomes, while interpersonal relationship adaptation may involve additional relational and contextual factors beyond individual proactive behaviors.

In addition to the hypothesized relationships, age and organizational tenure were included as control variables in the structural model to account for potential demographic differences among employees. As shown in Table 11, age categories (AGE1 and AGE2) did not significantly predict any dimensions of work adaptability, including performance proficiency, role clarity, interpersonal relationship adaptation, and cultural adaptation, as all bias-corrected bootstrap confidence intervals included zero. Similarly, most organizational tenure categories did not demonstrate significant effects on work adaptability. However, TEN2 showed a significant negative association with interpersonal relationship adaptation (β = −0.107, *p* = 0 .021, 95% BC CI [−0.200, −0.018]), indicating that employees in this tenure category reported lower levels of interpersonal relationship adaptation. Although TEN2 was negatively associated with cultural adaptation (β = −0.088, *p* = 0 .041), its bias-corrected confidence interval included zero (95% BC CI [−0.178, 0.008]), suggesting that this effect was not statistically robust. Overall, after controlling for age and organizational tenure, demographic characteristics showed limited explanatory power in predicting work adaptability.

## 5 Discussion

### 5.1 Discussion of findings

#### 5.1.1 Organizational socialization and work adaptability

This study provides a contextualized empirical extension of existing research by examining the associations among organizational socialization strategies, employee proactiveness, and multidimensional work adaptability within Chinese high-tech enterprises. Rather than treating work adaptability as a static organizational outcome, this study conceptualizes it as a multidimensional adjustment process linked to interactions between organizational contextual conditions and employee behavioral responses. Drawing on Organizational Socialization Theory, Social Cognitive Theory, and the Theory of Work Adjustment, this study explores how organizational environments relate with work adaptability within complex and changing workplace contexts.

The findings suggest that organizational socialization strategies are positively associated with work adaptability among employees in high-tech enterprises. Due to the characteristics of high-tech industries, including rapid technological iteration, intensive knowledge exchange, and continuously evolving work demands, employees often experience ambiguity regarding organizational expectations, job responsibilities, and interpersonal relationships. Organizational socialization strategies are related to greater understanding of organizational norms and lower perceived adaptation uncertainty, particularly in contexts where standardized informational resources, psychological support, and relational assistance are available. These findings align with the core assumptions of Organizational Socialization Theory, which proposes that structured organizational practices are related with employees’ acquisition of organizational knowledge and role clarity, both of which are relevant to successful organizational transition and workplace adjustment (Jones, 1986).

Among the three dimensions of organizational socialization strategies, social strategies showed the largest coefficient for employee proactiveness. One possible explanation is that social strategies may be particularly relevant in high-tech enterprises, where collaboration, knowledge sharing, and interpersonal interaction are important features of work. Through mentorship mechanisms, peer communication channels, and supportive communication climates, social strategies may provide informational and emotional support and may be associated with greater proactive participation in organizational affairs (Gong et al., 2026).

#### 5.1.2 Employee proactiveness and work adaptability

The findings further demonstrate that employee proactiveness serves as an important behavioral mechanism associated with work adaptability, which is consistent with the assumptions of Social Cognitive Theory (Bandura, 1982, 1986). Organizational environments are linked to employees’ motivational states, self-regulatory processes, and behavioral tendencies, which are subsequently reflected in behavioral outcomes. Organizational socialization strategies are characterized by supportive environments related to employees’ occupational confidence and psychological acceptance, which may in turn be associated with proactive behaviors such as information seeking, feedback inquiry, and work exploration.

These proactive behaviors are associated with employees’ acquisition of organizational knowledge, greater clarity regarding work requirements, and deeper organizational understanding, as well as with adaptation within complex work environments. Employee proactiveness exhibited positive associations with three dimensions of work adaptability: performance proficiency, role clarity, and cultural adaptation, but its direct association with interpersonal relationship adaptation was not statistically significant.

This result may reflect the different relational characteristics of the dimensions of work adaptability. Interpersonal relationships rely more heavily on reciprocal interaction, team acceptance, and supportive organizational climates (Kammeyer-Mueller and Wanberg, 2003; Schneider et al., 2013). Therefore, interpersonal integration may reflect not only individual proactive behaviors but also broader organizational social systems and team relational structures.

#### 5.1.3 The mediating role of employee proactiveness in the relationship between organizational socialization and work adaptability

The mediating role of employee proactiveness provides further insight into how organizational socialization strategies are associated with newcomer work adaptability. The findings indicate that organizational socialization strategies are not only directly related to adaptation outcomes but also show indirect associations with adaptation outcomes through employees’ proactive behavioral responses. This suggests that organizational practices provide contextual resources, while employees actively utilize these resources through information seeking, role adjustment, and adaptive behaviors.

From the perspective of Social Cognitive Theory, the findings highlight the reciprocal relationship between organizational environments and employee agency. Organizational socialization strategies are associated with supportive conditions and proactive behavioral responses, and these proactive behaviors are subsequently associated with adaptation outcomes. Therefore, employee proactiveness represents an important behavioral mechanism linking organizational socialization practices with newcomer adjustment in high-tech enterprises.

However, the mediation effects varied across different dimensions of work adaptability. Employee proactiveness demonstrated stronger mediating effects for performance proficiency, role clarity, and cultural adaptation, whereas its mediating effect on interpersonal relationships was weaker or unsupported in some paths. This finding suggests that proactive behaviors may be more effective in addressing work-related and cognitive adaptation demands, while interpersonal relationships may require additional relational resources, such as supervisor support, peer acceptance, and social network integration.

### 5.2 Theoretical contributions

At the theoretical level, this study contributes to the literature in three ways. First, it provides a contextualized empirical extension of existing research on organizational socialization, employee proactiveness, and multidimensional work adaptability within Chinese high-tech enterprises. Examining these relationships in a knowledge-intensive organizational context offers further insights into the associations between organizational socialization strategies and newcomer adaptation outcomes.

Second, this study extends previous research by examining the behavioral mechanism of employee proactiveness in the relationship between organizational socialization and work adaptability. The findings suggest that organizational socialization strategies are associated with adaptation outcomes partly through employees’ proactive behaviors, highlighting the role of individual agency in utilizing organizational resources during newcomer adjustment.

Third, this study advances the understanding of work adaptability as a multidimensional construct by demonstrating that employee proactiveness does not uniformly relate to all adaptation dimensions. Specifically, employee proactiveness was positively associated with performance proficiency, role clarity, and cultural adaptation, but not interpersonal relationships. This finding suggests that work-related and cognitive adaptation may rely more strongly on proactive behaviors, whereas interpersonal relationships may require additional relational resources, such as supervisor support, peer acceptance, and social integration opportunities.

### 5.3 Practical implications

The findings provide several practical implications for high-tech enterprises. First, organizations should adopt structured organizational socialization strategies, such as clear onboarding, role clarification, and timely organizational support, to facilitate newcomer adjustment. Second, managers should foster employee proactiveness by encouraging feedback seeking, continuous learning, and open communication. Third, mentoring and supportive workplace relationships should be strengthened to facilitate newcomers’ social integration and work adaptation. As organizational adjustment is a continuous process in high-tech enterprises, HR managers should provide ongoing developmental support beyond the initial onboarding stage. These practices may be associated with newcomer work adaptability and may have implications for longer-term employee retention.

### 5.4 Limitations and future research

This study has several limitations. First, the sample was limited to three high-tech enterprises in Jinan, which may restrict the generalizability of the findings. Future research may extend the sample across different regions, organizational contexts, and industries to enhance external validity.

Second, this study defined newcomers as employees with organizational tenure of 3 years or less. Although this criterion is consistent with prior research, employees within this range may experience different stages of adjustment. Future studies may adopt more refined tenure criteria and further examine the robustness of the findings across different stages of newcomer adjustment.

Third, this study adopted a cross-sectional design, which limits the ability to examine temporal changes and establish causal relationships among organizational socialization strategies, employee proactiveness, and work adaptability. The findings should therefore be interpreted as theoretically informed associations and mediation relationships rather than evidence of developmental processes over time. Future research may employ longitudinal designs to further investigate how work adaptability develops and changes across different stages of organizational entry and adjustment.

Fourth, employee proactiveness was treated as a single construct. Future studies may further examine different dimensions of proactive behavior and incorporate additional moderating variables, such as leadership style and team climate, to provide a more comprehensive understanding of work adaptability mechanisms.

Fifth, the study relied primarily on self-reported questionnaire data collected at a single point in time. Although procedural and statistical approaches were employed to assess common method bias, the possibility of residual method-related bias cannot be completely excluded. Future research could combine employee self-reports with supervisor-rated performance or objective indicators and adopt multi-wave designs to strengthen the robustness of the findings.

### 6 Conclusion

The results suggest that organizational socialization strategies are positively associated with employee proactiveness, which in turn is positively related to performance proficiency, role clarity, and cultural adaptation. Furthermore, Employee proactiveness significantly mediated several relationships between organizational socialization strategies and work adaptability, particularly those involving performance proficiency, role clarity, and cultural adaptation.

Overall, this study highlights the important role of employee proactiveness in the organizational socialization process and provides empirical evidence regarding the potential mediating role of employee proactiveness underlying the relationships between organizational socialization strategies and employees’ work adaptability. By doing so, the study contributes to the existing literature on organizational socialization and work adaptability. Moreover, the findings offer important theoretical and practical implications for organizations seeking to refine newcomer socialization practices and support employees’ long-term adaptation and organizational integration.

## Data Availability

The original contributions presented in the study are included in the article/Supplementary material, further inquiries can be directed to the corresponding author.
