# Correction: Organizational socialization and new employees' work adaptability in high-tech enterprises: the mediating role of employee proactiveness

**DOI:** 10.3389/fpsyg.2026.1998912

**Published:** 2026-10-08

**Authors:** Shuhua Liu, Chin-Hong Puah

**Affiliations:** 1 Faculty of Economics and Business, Universiti Malaysia Sarawak, Sarawak, Malaysia; 2 Business School, Qilu Institute of Technology, Jinan, Shandong, China

**Keywords:** employee proactiveness, high-tech enterprises, mediating effect, organizational socialization, work adaptability

In the published article, Affiliation 2 was erroneously written as:

Business School, Qingdao Institute of Technology, Jinan, Shandong, China

The correct affiliation is:

Business School, Qilu Institute of Technology, Jinan, Shandong, China

The original version of this article has been updated.

